# Advances in the Study of Marine Products with Lipid-Lowering Properties

**DOI:** 10.3390/md18080390

**Published:** 2020-07-27

**Authors:** Jiarui Zhao, Qi Cao, Maochen Xing, Han Xiao, Zeyu Cheng, Shuliang Song, Aiguo Ji

**Affiliations:** 1 Marine College, Shandong University, Weihai 264209, China; 201936684@mail.sdu.edu.cn (J.Z.); sddxcqq@163.com (Q.C.); sddxxmc@163.com (M.X.); 15651795075@163.com (H.X.); kuerk18686@163.com (Z.C.); 2 School of Pharmaceutical Sciences, Shandong University, Jinan 250012, China

**Keywords:** cardiovascular diseases, atherosclerosis, hyperlipidemia, hypolipidemic, marine products

## Abstract

With twice the number of cancer’s deaths, cardiovascular diseases have become the leading cause of death worldwide. Atherosclerosis, in particular, is a progressive, chronic inflammatory cardiovascular disease caused by persistent damage to blood vessels due to elevated cholesterol levels and hyperlipidemia. This condition is characterized by an increase in serum cholesterol, triglycerides, and low-density lipoprotein, and a decrease in high-density lipoprotein. Although existing therapies with hypolipidemic effects can improve the living standards of patients with cardiovascular diseases, the drugs currently used in clinical practice have certain side effects, which insists on the need for the development of new types of drugs with lipid-lowering effects. Some marine-derived substances have proven hypolipidemic activities with fewer side effects and stand as a good alternative for drug development. Recently, there have been thousands of studies on substances with lipid-lowering properties of marine origin, and some are already implemented in clinical practice. Here, we summarize the active components of marine-derived products having a hypolipidemic effect. These active constituents according to their source are divided into algal, animal, plant and microbial and contribute to the development and utilization of marine medicinal products with hypolipidemic effects.

## 1. Introduction

According to the “2020 World Health Statistics report” [1] 41 million people worldwide die of non-communicable diseases (NCDs). 71% of all deaths, i.e., 17.9 million people die from cardiovascular diseases, twice the number of cancer’s deaths and approximately one third of all global deceases. With this cardiovascular diseases (CVD) stand as the leading cause of deaths worldwide. CVD is the name for the cluster of disorders afflicting the heart and blood vessels, including hypertension (high blood pressure), coronary heart disease (heart attack), cerebrovascular disease (stroke), heart failure and peripheral vascular disease [2]. Interestingly, in high-income countries, cancer is the leading cause of premature deaths while in other countries, particularly with low and low to middle income, CVD continue to be the main NCD cause that claims the largest number of lives among people between ages 30 and 70 [1].

Characterized by gradual accumulation of lipid and inflammatory cells in the intima of arteries, atherosclerosis, a chronic inflammatory disease of large and medium-sized arteries, is a common cause of CVD, which occurs within arterial vasculatures predominantly at sites of disturbed flow or localized changes in blood rheology, such as at the site of vessel bifurcation where lipids and apoB-lipoproteins are much easier to accumulate [3,4,5]. It is a progressive disease caused by persistent damage to blood vessels triggered by elevated cholesterol levels. Although myocardial infarction occurs much more quickly, advanced, complex inner wall lesions causing plaque rupture in coronary heart disease can take decades. Due to the long course of atherosclerosis, the risk of related diseases increases exponentially with age [6]. Hyperlipidemia is an important reason for atherosclerosis [7]. Studies have shown that the risk of coronary heart disease is positively correlated with the level of low-density lipoprotein cholesterol (LDL-C) and negatively correlated with the level of high-density lipoprotein cholesterol (HDL-C) [8]. Even for adults with low risk of cardiovascular disease, hyperlipidemia is positively correlated with the risk of coronary heart disease in their 40 s and 50 s [6].

With the change of living habits and improvement of living standards, the high-fat diet has become increasingly common, followed by a gradual increase in the frequency of hyperlipidemia, which is usually manifested by elevated serum total cholesterol (TC), triglyceride (TG) and low-density lipoprotein (LDL) levels, and low levels of high-density lipoprotein (HDL). It leads to a variety of diseases and is a usual cause of CVD. A high-fat diet encourages people to eat more because of its high energy density, palatability and lower satiety than that of protein and sugar, all of which contribute to the body’s fat gain [9,10,11]. Generally, the steady state of lipid content plays an important role in life, such as releasing energy through oxidative decomposition. It also participates in vitamin absorption, regulates cell transmembrane transport and contributes to hormone synthesis. Disturbed cholesterol balance underlies not only CVD but also an increasing number of other diseases such as neurodegenerative illnesses and cancers [12].

At present, studies have shown that the long-term use of lipid-lowering drugs improves the survival status of patients with coronary heart disease though to a certain extent [13]. Generally, the appropriate use of some drugs to control the level of blood lipids, mainly for the improvement of living standards has important significance. Among the lipid-lowering drugs used in the clinic, statins are recognized as the cornerstone of pharmacological treatment for reducing the levels of LDL cholesterol. Statins are inhibitors of 3-hydroxyl 3-methyl glutaryl coenzyme A reductase (HMGCR), which controls the rate-limiting step in the cholesterol biosynthetic pathway, inhibition of which reduces hepatic cholesterol and up-regulates the liver LDL receptor (LDLR) [14]. In addition to lovastatin, which is the first drug to hit the market, there are six other statins available to date: simvastatin, pravastatin, fluvastatin, atorvastatin, rosuvastatin and pitavastatin. Large randomized controlled trials, including the landmark 4S (Scandinavian Simvastatin Survival Study) [13], WOSCOPS (West of Scotland Coronary Prevention Study) [15], and HPS (Heart Protection Studies) [16] have shown that simvastatin therapy decreases morbidity and mortality in patients with atherosclerotic vascular disease while pravastatin improves the living conditions in people at high risk of nonfatal events of atherosclerosis with high levels of LDL-C [17]. Despite the benefits of statins for cardiovascular disease, the side effects are common such as the most common statin-related muscle symptoms: myalgia, myositis and rhabdomyolysis as well as liver damage [18].

Current drug development is inseparable from the exploration of new bioactive substances and marine organisms are the most valuable natural resources of active substances [19]. Many epidemiological studies have found that the incidence of obesity-related diseases is low among people eating marine products, indicating that marine products have certain beneficial effects [20]. At present, many marine compounds have been found to have lipid-lowering activities and have the prospect of drug development [21]. In this paper, some marine products with lipid-lowering effect are selected to be reviewed to promote the development and utilization of related bioactive substances.

**Figure 1:**
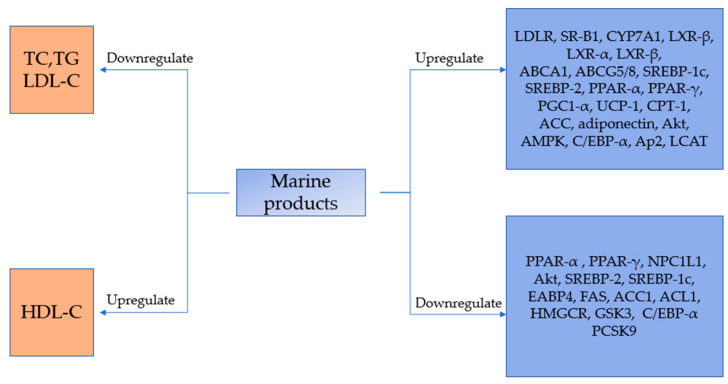
The hypolipidemic effect of marine products and the possible mechanism of it, which can be different between cell lines.

We have prepared an icon showing the marine products with lipid-lowering effects and their possible mechanism, as shown in Figure 1.

## 2. Algal Marine Products

Among many marine products, algae are important sources of compounds with diverse structures and bioactivities. Seaweeds, also known as macroalgae, growing on the bottom of relatively shallow coastal waters, are important biological resources. They systematically are divided into three major phyla Chlorophyta (green algae), Phaeophyta (brown algae) and Rhodophyta (red algae). Seaweed provides key nutrients such as saccharides, proteins and minerals [22,23]. Many studies on the hypolipidemic effect of seaweed extracts exist. For example, researchers [24] have found that *Grateloupia elliptica* extract (GEE), a red seaweed from Jeju Island in Korea, exerted hypolipidemic activity in 3T3-L1 cells and in mice on a high-fat diet (HFD), significantly reducing the expression of adipogenic proteins, sterol-regulatory element binding protein (SREBP)-1 and peroxisome proliferator-activated receptor (PPAR)-gamma while enhancing the expression of metabolic regulator protein in white adipose tissue (WAT). It has been further shown that PPAR gamma and CCAAT/enhancer binding protein (C/EBP) alpha mRNA levels were downregulated in WAT, while the expression of thermogenic proteins in brown adipose tissue were upregulated [24]. Others have shown that *Sargassum liebmannii* reduced energy intake, improved insulin sensitivity and as a result reduced adipose tissue content in rats [25]. Although the specific mechanism of their lipid reducing effect is yet unknown, these preliminary studies have laid a solid foundation for future research on specific active molecules and their underlying mechanisms of action.

### 2.1. Seaweed Polysaccharides

Biodegradable, water-soluble and functional seaweed polysaccharides are biological macromolecules in the cell wall structure of marine algae, though they are not widely exploited and utilized [26]. Sulphated polysaccharides are complex heterogeneous natural polymers found abundantly in different marine algae species. Their chemical composition and structure vary with the different extraction sources and methods. The presence and quantitative contents of carbohydrates, proteins, sulphates and the degree of sulphation yield different pharmacological effects, such as antioxidant, antiallergic, antiviral, anticancer, anticoagulation, cholesterol-reducing, anti-free radicals and heart protective roles. The range of application is wide and spans from the food, cosmetic and pharmaceutical industry to microbiology and biotechnology [27,28]. Porphyran, sulphated polysaccharide extracted from the marine red algae *Pyropia yezoensis*, can reduce the blood serum levels of TG, TC, LDL-C/HDL-C ratio, whereas improves fecal TC and TG levels, and increases liver injuries induced by HFD [29]. A research discussed the effects of sulphated polysaccharides from the edible seaweed *Padina tetrastromatica* (PSPS) in myocardial infarction rat model induced by isoproterenol (ISO), which shows that PSPS adjusts the expression of SREBP-2 and LDLR, maintains the steady state of lipids content, improves oxidative stress in rats, the endothelial dysfunction and soothes inflammation caused by ISO treatment. The overall effect of PSPS is comparable to the reference drug aspirin, revealing the fact that PSPS reduces cardiovascular complications [30]. Better understanding of the relationship between the physical and chemical properties and the biological activity of these seaweed polysaccharides is essential for successful application and exploration of their additional functions [26].

Familiar polysaccharides from algae include fucoidan from brown algae, carrageenan from red algae and ulvan from green algae and will be discussed further.

#### 2.1.1. Fucoidan

**Figure 2:**
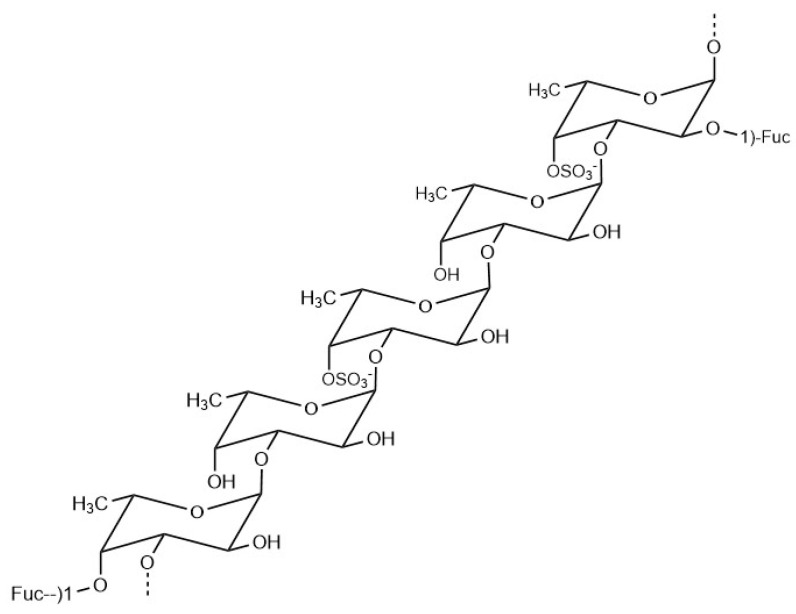
General structure of fucoidan from Fucus vesiculosus.

Fucoidans are a series of complex sulphated polysaccharides existing in the cell walls of large brown algae. Its chemical composition and structure are very complicated and vary from species to species [31]. Fucoidan extracted from *Fucus vesiculosus* is commercially available at present, with a chemical structure shown in Figure 2, mainly composed of alpha-(1→3) linked fucose polymers with sulphate groups substituted at the C-4 position on some of the fucose residues. At the same time, there is a fucose connected to the polymer to form a branch point every 2 or 3 residues within the chain [31]. Studies have shown that the activity of fucoidan increases with the degree of sulphation [32]. Studies have shown that fucoidans have a variety of physiological functions, such as lipid-lowering, anti-tumor [33], anti-virus [34], anti-aging [35], immune regulation [36], hypoglycemic activities [37], and so on.

Researchers have recently discovered that fucoidan A3, extracted from the brown algae Ascophyllum nodosum significantly reduces the blood serum levels of TC, TG and the fat pad index in hyperlipidemic mice [38]. It was also shown that it boosts the mRNA expression of several important genes like LDLR, the scavenger receptor (SR)-B1, the cholesterol 7 alpha-hydroxylase A1 (CYP7A1), the liver X receptor (LXR)-beta, the ATP-binding cassette transporter (ABC)A1 and SREBP-1c. It was shown that it decreases the expression of PPAR gamma, upregulating lipid transfer from the plasma to the liver, thus improving the lipid metabolism and exhibiting anti-hyperlipidemic activity [38]. 

Furthermore, others showed that fucoidan from the brown alga Ascophyllum nodosum improves the lipid levels of ApoE (-/-) mice and increases the expression of SR-B1, PPAR-alpha, LXR-alpha, ABCA1 and ABCG8 in the liver [39]. The mRNA and protein expression of PPAR, SREBP-1c and the Niemann-Pick C1 like 1 (NPC1L1) were significantly reduced while the level of ABCG8 was significantly increased, indicating that fucoidan plays a lipid-lowering role by regulating the expression of RCT-related genes [39]. Other authors have shown that fucoidan from the brown algae *Fucus evanescens* normalizes the key parameters of lipid metabolism [40]. By the way, another study has demonstrated that fucoidan reduces serum levels of TC, TG and LDL cholesterol, and increases HDL levels in hyperlipidemia mice induced by poloxamer-407 [41]. It reduces the expression of FAS and ACC in HepG2 cells without or only slight inhibitory effect on SREBP-1c mRNA expression. Data show that fucoidan attenuates the hepatic expression of mature SREBP-2 protein with a subsequent decrease in HMGCR mRNA expression levels and with an increase in hepatic LDLR mRNA expression. In addition, fucoidan also alleviates the atherosclerotic lesions in the aorta of mice [41]. The complex chemical structure makes it quite challenging to find a specific structure-activity relationship. Though with the technological progress and continuous exploration, the understanding of the specific mechanisms of action of fucoidan will be further deepened, which is a condition for its application in the pharmaceutical and food industries.

#### 2.1.2. Alginate

Alginate is a natural polysaccharide composed of guluronic (G) and mannuronic acid (M) and is one of the structural components of brown algae. A study showing that sodium alginate from *Turbinaria ornata (Turner) J. Agardh* dose-dependently reduced TC and LDL-C levels and increase HDL-C levels in alloxan-induced diabetic mice just have been conducted [42]. Alginate can significantly reduce liver cholesterol concentration, but has little influence on serum cholesterol, triacylglycerols, and total hepatic lipids, whereas amidated alginate significantly reduces serum cholesterol, TG levels, total hepatic lipids and cholesterol [43]. Some authors [44] have demonstrated that calcium alginate effectively reduces the concentration of plasma cholesterol due to reduced intestinal reabsorption and increased excretion of bile acid in feces, stimulating the transformation of cholesterol in the liver into bile acid and lowering the plasma cholesterol levels. Though the lipid-lowering effect is significant, the specific mechanism is yet unclear. At the same time, some studies show that the ability to suppress coagulant factors activities and weak platelet function of alginate with low mannuronic acid/guluronic acid ratio and high molecular weight brings about bleeding, making it necessary to control relevant parameters [45]. Above all, relevant work still needs the continuous efforts of scientific researchers.

#### 2.1.3. Ulvan

Ulvan is heteropolysaccharide composed mainly of rhamnose, xylose, glucose, glucuronic acid, iduronic acid and sulphate with smaller amounts of mannose, arabinose and galactose [2]. In Asia, *Ulva pertusa* is a marine-derived vegetable, which is rich in vitamins, oligomers, minerals and dietary fibers [46]. A study showed that ulvan from green algae *Ulva pertusa* considerably reduces the TC, TG and LDL-C levels of mice fed with a high-fat diet (HFD) and increases HDL-C blood serum levels, showing a great lipid-lowering effect [47]. Pristine ulvan and phosphorylated one significantly reduced the activity of major liver oxidases such as glutathione peroxidase (GSH-Px), superoxide dismutase (SOD) and catalase (CAT) and decreased the weight of mice fed with a high-fat diet, thus lowering the serum concentration of LDL-C, TC and TG significantly and increasing the level of HDL-C [48]. The heterogeneity of ulvan has brought great challenges to the study of its related properties. With the time, the number of studies on the effects and mechanisms of action of ulvan have gradually increased, which is good for the deep understanding of lipid-lowering activity of ulvan, all of which are conducive for its promotion and application in the practice.

#### 2.1.4. Carrageenan

Carrageenan is a linear high molecular weight polysaccharide, which contains repeated galactose units and 3,6-anhydrogalactose (3,6 AG), linked by alternating alpha-(1,3) and beta-(1,4) glycosidic bonds [2]. Eucheuma spinosum (Rhodophyceae) is used for the commercial production of carrageenan [2]. Data show that carrageenan from *Kappaphycus alvarezii* could reduce the serum cholesterol level, the size of the adipose tissue and the concentration of adipose factor, thus modulating gut dysbiosis caused by HFD in mice [49]. Some authors demonstrated that the low molecular weight carrageenan significantly reduces blood serum levels of TC, TG and LDL-C and improves the concentration of HDL-C in HFD fed mice, with a much stronger activity than that of high molecular weight carrageenan [50]. Furthermore, a considerable number of studies have shown that carrageenan causes inflammation [51,52,53], which should be fully considered in further studies, as this puts forward some limitations on the risk-free application of carrageenan. Although with certain side effects, further in-depth research on molecular mechanisms will help the development and utilization of Carrageenan.

In addition, some small molecular active substances with lower molecular weight such as carotenoids and polyphenols will be discussed later.

### 2.2. Fucoxanthin

**Figure 3:**
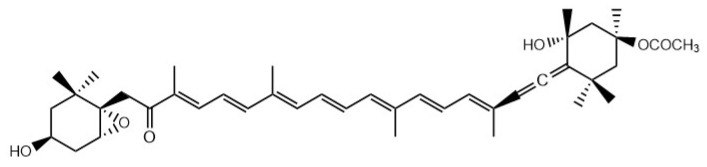
The structure of fucoxanthin.

Fucoxanthin is an oxygen-containing carotenoid isolated from brown algae [54]. Its chemical structure is shown in Figure 3 [55]. Apart from the bioactivities like anti-tumor [56] and anti-aging effect [57], researchers [58] have shown that fucoxanthin from *Undaria pinnatifida* increases the activity of major players in energy consumption like the PPAR alpha, the PPAR-gamma coactivator 1α (PGC1 alpha) and the PPAR gamma and uncoupling protein (UCP)-1. The treatment augmented the fat beta oxidation, amended the acetyl-CoA carboxylase (ACC) gene expression, increased the plasma concentration and expression of adiponectin and reduced fat accumulation and lowered plasma levels of cholesterol and TG, ultimately reducing lipid accumulation. Some studies show that fucoxanthin reduces the body weight and the epididymal fat weight in diabetic mice, upregulates the expression of AKT and Adenosine 5′-monophosphate-activated protein kinase (AMPK), downregulates the expression of GSK3, reduces plasma TG and TC levels, and improves lipid metabolism in diabetic mice [55]. Others have shown that fucoxanthin reduces body fat in mice induced by HFD, decreases lipid uptake and turnover in visceral WAT while upregulating the expression of related genes key to fatty acid oxidation and thermogenesis (CPT1, UCP1) in subcutaneous WAT and enhances UCP1 protein in interscapular brown adipose tissue to ease the accumulation of fat caused by HFD [59]. Fucoxanthin’s ability to reduce body weight, BMI and abdominal fat and to improve body fat status by acting on visceral and subcutaneous fat is shown in the research conducted [60]. To some extent, fucoxanthin is excellent because there are already many commodities with it as the main component. With the developing understanding of its specific mechanisms of action, the application of fucoxanthin promises broader applications.

### 2.3. Phlorotannins 

Found in some brown algae, phlorotannins are phenolic compounds formed by the polymerization of phloroglucinol or 1,3,5-trihydroxybenzene monomer units [61]. Recent data show that the phlorotannin-rich extract extracted from *Ecklonia cava* diminishes blood serum levels of TC and LDL-C without changing the amount of TG and HDL-C [62]. Other studies [63] show that diphlorethohydroxycarmalol (DPHC) extracted from brown algae *Ishige okamurae* is able to reduce adiposity and body weight gain in mice fed with HFD. DPHC was shown to reduce the serum levels of TG, LDL-C, leptin and aspartate transaminase as well as to improve the level of serum HDL-C. It significantly inhibits the lipid accumulation in the liver by downregulating the expression of the critical enzymes for lipogenesis including SREBP-1c, FABP4, and FAS, as well as decreases the expression of lipogenic proteins and enzymes including PPAR gamma, C/EBP alpha, SREBP-1c, FABP4, and FAS in the epididymal adipose tissues (EAT). Furthermore, DPHC stimulates the phosphorylation of AMPK and ACC in the liver and EAT, demonstrating hypolipidemic properties [63].

Algal marine products such as the above-discussed polysaccharides, carotenoids and phlorotannins demonstrate well-proven ability to improve dyslipidemia in cellular and animal models through various mechanisms. Some of their activities lead to lowering of blood serum levels of cholesterol, thus presenting good options for developing these compounds as materials for human drug development. Because of the difficulty for their structure detection, bioavailability, dosage and side effects, further research on the mechanism of their action is still needed for the development and utilization of these bioactive substances in the practice.

We have organized the algae-derived products with hypolipidemic effects in recent years, as shown in Table 1. 

## 3. Marine Products of Animal Origin

The majority of marine products of animal source originate from a huge number of edible fishes. They are abundant in proteins, bioactive peptides, lipids and polysaccharides and are excellent readily available source of lipid-lowering compounds. There have been many studies on these substances, some of which have been fully applied as health products, and achieved gratifying results. Even though the degree of exploitation and utilization of marine resources is currently yet relatively low, expecting extensive studies on their exact mechanisms of action before any broad utilization in practice.

### 3.1. Proteins and Bioactive Peptides

Recently, the acquisition, characterization and application of proteolytic and food-derived biopeptides have aroused great interest due to their numerous health-beneficial effects [68]. It is well-known that the bioavailability of oral therapeutic peptides depends on their ability to cross the intestinal epithelial barriers and reach the systemic circulation [69]. Despite poor intestinal permeability, peptides of different sizes can still pass through intestinal epithelial barriers through active or passive (paracellular or transcellular) transport pathways [70]. A small number of bioactive peptides penetrating the intestinal barrier have strong biological activity [71]. In addition, lipidation of peptides increases the lipophilicity, enhancing the intestinal membrane permeability by modulation of tight junctions [72]. These proteins are bioactive nutrients in fish that can affect lipid metabolism and demonstrate cardiovascular protection. Recent data show that subcritical water-hydrolyzed fish collagen peptide can significantly inhibit lipid accumulation in 3T3-L1 pre-adipocytes during differentiation, and decrease gene expression levels of C/EBP-alpha, PPAR-gamma and adipocyte protein 2 (aP2) [73]. In addition, it was found that it inhibits palmitate-induced accumulation of lipid vesicles in hepatocytes. In animal experimental models, i.e., epididymal adipose tissue of mice fed HFD, this peptide significantly reduced serum levels of TC, TG, and LDL, increased the serum HDL levels, and inhibited the expression of C/EBP-alpha, PPAR-gamma, and aP2, thus resulting in a significant reduction of the adipocyte size [73]. The goby protein hydrolysates (GPH) can significantly diminish blood serum levels of TC, TG and LDL-C in rats fed with a high-fat diet, suggesting that it plays a lipid lowering role by inhibiting lipogenesis, lipid absorption and digestion, thus preventing the risk of coronary artery disease [74]. GPH inhibits the activity of pancreatic lipase, which degrades dietary TG into monoacylglyceride and free fatty acids, reducing digestion and absorption of lipids by intestinal cells, upregulating the excretion of lipid into feces, and thus improving dyslipidemia [74]. Data show that sardine protein reduces the levels of TC and TG in the liver and in the blood serum of rats fed with HFD, improves the excretion of cholesterol in feces, increases the activity of lecithin cholesterol acyltransferase (LCAT), and improves the reverse transport of cholesterol [75]. The protein hydrolysates and protein powder of *Sardinella aurita* could significantly reduce TG, TC and LDL-C blood serum levels, consequently increasing HDL-C levels in rats fed with a high-fat diet. Among them, protein hydrolysates had a better effect, not only by reducing TC and TG levels in liver, but also decreasing the pancreatic lipase activity. Both of them reduced the adipose tissue weight in rats [76]. Some authors [77] have demonstrated that the visceral proteolysis of starfish reduces TC and TG levels and improves lipid metabolism in HFD rats. Another research show that hydrolysates of *Octopus vulgaris* muscles proteins decreases the levels of TG, TC and LDL-C in hyperglycemia rats induced by alloxan, improves the lipid metabolism as well as reverses the increase in the activities of alanine aminotransferase (ALT), aspartate aminotransferase (AST), alkaline phosphatase and gamma-glutamyl transpeptidase caused by alloxan, playing a certain role in liver protection [78]. All these data prove without appeal that marine bioactive peptides from animal sources have a good prospect for their application in the development of lipid lowering drugs. Therefore, insisting on the need for further research on the structure and structure-activity relationship of specific bioactive marine animal peptides. Given the fact that some biopeptides may have a certain degree of biotoxicity, the effect of toxicity on cellular and animal models should be taken into consideration.

### 3.2. Lipids

Epidemiological studies have shown that fish oil (FO) is rich in N-3 polyunsaturated fatty acids (PUFAs) and successfully reduces the risk of cardiovascular pathologies such as cardiac death and myocardial infarction through anti-inflammatory, anti-atherogenic and antithrombotic pathways [79]. Long chain omega-3 fatty acids (FAs) effectively reduce plasma TG levels. Animal and human experiments have additionally proved the ability of FO to reduce TG. It was further discovered that this activity is due to the key ingredients of FO, i.e. the docosahexaenoic (DHA) and eicosapentaenoic acid (EPA). As is shown in the well-known Reduction of Cardiovascular Events with Icosapent Ethyl-Intervention Trial (REDUCE-IT) [80], icosapent ethyl, a purified formulation of EPA, can appreciably lower the CVD risk in patients treated with statin. There was a 25% relative risk reduction (RRR) and 4% absolute risk reduction in the primary CVD end point (cardiovascular death, nonfatal myocardial infarction, stroke, revascularization and hospitalization for recurrent angina) with a number needed to treat of 21 to prevent one event over a 5-year period, showing that icosapent ethyl can become a safe and effective therapy for patients with hypertriglyceridemia at increased CVD risk [81]. Though the TG-lowing effect was modest, there must have other pleotropic mechanisms to be discussed contributing to the clinical benefits, one of which may be improving endothelial function when combined with statin therapy [82]. Others like attenuating expression of inflammatory genes and producing HDL particles may also contribute to the benefits of EPA [83,84,85].

FO suppresses intracellular lipolysis in adipocytes by suppressing adipose tissue inflammation. It also increases extracellular lipolysis exerted by lipoprotein lipase (LPL) in adipose, heart and skeletal muscles and enhances hepatic and skeletal muscle beta-oxidation, reducing fatty acids it the liver [86]. Although the effect of FO on TG is clear, the specific mechanism of its action is hitherto unknown. One potential mechanism can be an inhibition of the expression of lipogenic genes, suppression of key enzymes involved in liver TG synthesis and an increment in beta-oxidation of fatty acids and induction of the expression of the lipoprotein lipase, which in turn contributes to the lipid-lowering effects [87]. Recent results have shown that FO significantly reduces the plasma TC, TG and free fatty acid levels in HFD mice [87]. In addition, deep-sea fish oil significantly decreases the serum levels of TC, TG and LDL-C in HFD rats, reduces the atherosclerosis index (Al) and increases the protein expression of liver SIRT1 and PPAR-alpha [88]. Furthermore, there are data showing that the starfish oil dose-dependently improves hyperlipidemia and liver lipid accumulation in mice fed HFD, and improves liver metabolism disorders [89]. The EPA-rich phosphatidylcholine and phosphatidylserine extracted from the sea cucumber *C. frondosa* expressively inhibits the expression of SREBP-1c in the liver and thus reduces lipogenesis, activates the beta-oxidation of fatty acids by improving the expression of PPAR-alpha and thus plays a strong hypolipidemic role [90]. 

Here, we stress on the point that the future successful application of the above-presented bioactive lipid compounds found in marine animal products cannot be separated from the deep exploration on its mechanisms of action. Therefore, further detailed research on marine oil is a prerequisite for a solid foundation for its application in the food and pharmaceutical industries and in health care.

### 3.3. Polysaccharides

In addition to the abovementioned algal polysaccharides, marine animal-derived polysaccharides exhibit significant biological activity. Researches have shown that the sulphated polysaccharides of the abalone gonad (AGSP) reduce the serum levels of TC, TG and LDL C, optimizes the level of HDL-C and downregulates the concentration of liver TC, TO and MDA [91]. It also optimizes the gut microbiota dysbiosis, inhibits fat tissue GPR43 gene expression and raises the expression of GPRA41, which in turn inhibits lipid accumulation of epididymal adipose tissue in HFD fed mice [91]. Data show that mice fed with HFD show that sulphated polysaccharides from *Stichopus japonicus* reduce body weight, decrease serum lipid levels, prevent HFD-induced intestinal disease as well as optimize endotoxin levels. These findings indicate that polysaccharides can regulate intestinal microbiota and tissues to prevent obesity and related diseases caused by diet, which can be improved by free-radical depolymerization [92].

#### 3.3.1. Fucoidans

**Figure 4:**
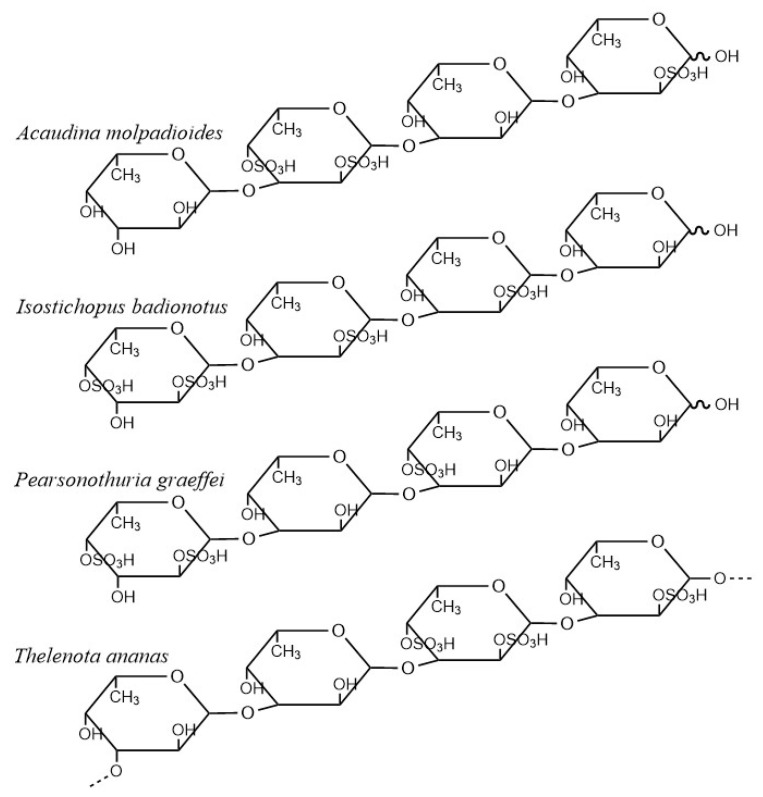
The structure of some fucoidans from sea cucumbers.

Fucoidans from the brown algae are usually heterogeneous with complex chemical composition and side chains, which makes it difficult to understand the relationship between the structure and the exhibited biological activity. Different from the fucoidans of brown algae, the fucoidans from marine invertebrates are often composed of linear structures with uniform repeating units, which allows the study of the relationship between structure and function [93]. Fucoidans from sea cucumbers, for example, have uniform and repetitive tetrasaccharide units, linked by alpha-1, 3-glycoside bonds. The difference of these fucoidans lies in the sulphation site, namely, 2-O-,4-O-and 2, 4-O-sulfonic substitutions, as is shown in Figure 4 [94,95].

Recent research shows that the fucoidan extracted from *Pearsonothuria graeffei* and *Isostichopus badionotus* reduces the weight and the serum TG levels of rats exposed to a high-fat diet. Among them, fuc-PG reduces TC and LDL-C, increases HDL-C levels and adiponectin levels, reverses the increased levels of CD36, PPAR alpha and CYP7Al in rats caused by HFD, and improves lipid metabolism disorder, while fuc-IB has no effect on the abnormal levels of cholesterol and adiponectin. In addition, fuc-PG significantly reduces ALT and AST levels and reduces total bile acid levels, showing a protective effect on the liver, while the hepatoprotective effect of fuc-IB is relatively weak [93]. The difference between the two fucoidans lies in the fact that fuc-PG is mainly composed of 4-O-sulfation, while fuc-IB is mainly composed of 2-O-sulphation. The results show that the presence of different sulfation sites have a certain influence on the activity of fucoidan. For example, fucoidan dominated by 4-o-sulfation shows better lipid lowering effect [93]. Besides, researchers [96] also studied the structure-activity relationships of four well-defined sulphate polysaccharides obtained from the sea cucumber, and the results showed that fucosylated chondroitin sulphate and fucoidan with a higher degree of linearity showed great hypolipidemic effect while fucoidan with a lower degree of linearity showed a poorer effect. These results indicate that the structure characteristics including side chain and sulphation pattern could influence the chain conformation of polysaccharides, thus determining their physical and chemical properties and lipid-lowering activities. The relatively ordered structure brought considerable convenience for studies on the structure-activity relationship of fucoidans and is promising for its application in practice.

Apart from fucoidans, chitosan is much more famous and will be discussed later.

#### 3.3.2. Chitosan

A well-known marine polysaccharide with animal origin is chitosan, which has a variety of physiological functions like lipid-lowering, drug delivery [97], anti-aging [98], immune regulation [99], hypoglycemic activities [100]. It is the most abundant renewable organic resources in the world and is a major component of crustacean shells, insect exoskeletons, and fungal cell walls. Chitosan provides strength and stability, and its synthesis and degradation in the biosphere is estimated to reach more than 10 G tons per year. On the chemical composition, chitosan is composed of (1→4) linked 2-acetyl amino -2-deoxidation-beta-d-glucose unit (or acetyl-d-glucosamine) with a long chain of linear polymers [101]. According to the sources, chitosan has different degrees of N-acetylation, which comprises acetyl -d-glucosamine and d-glucosamine copolymers, making the chitosan molecule look like the cellulose. Generally, chitosan is insoluble in water and in most ordinary solvents, but the presence of amino groups makes it solvable in acidic solutions with pH around 6.5. Chitosan is not a monomer compound and composition changes with the sources and synthesis process. Studies showed that chitosan could significantly reduce the serum levels of TG, LDL and TC and in the liver of mice fed with a high-fat diet. It was shown to reduce the expression of CD36, PXR, DGAT2, LXR alpha and PPAR gamma, and to downregulate fatty acid uptake and TG synthesis as well as to lower the liver steatosis, consequently altering lipid accumulation [102]. Research by other authors showed that chitosan can significantly reduce TC, TG and LDL-C blood serum levels and increase the HDL-C blood serum levels in rats. It was further demonstrated that chitosan optimizes the activity of LPL, hepatic lipase, SOD and glutathione peroxidase as well as lowers lipid accumulation while downregulating the levels of ALT and AST, which in turn protects the liver [103]. Some researchers have established cellular and animal models to study the effects of chitosan on hyperlipidemia and have shown that chitosan considerably reduced lipid accumulation in HepG2 cells and the expression of HMGCR, SET and MYND domain containing 3 (SMYD3) in animal models [104]. It significantly reduced the serum levels of TC, TG and LDL-C in diabetic mice, raised the expression of CYP7A1, lowered the expression of SMYD3 and the synthesis of cholesterol, as well as accelerated the metabolism of cholesterol. Furthermore, it was shown that it reduces the levels of liver inflammation, having hepatoprotective effect to some extent. Subsequent experiments proved that chitosan could down-regulate the expression of SMYD3, through which cuts down the expression of HMGCR and activates CYP7A1, thereby exerting its hypolipidemic effect [104]. The above-listed beneficial effects of chitosan attract the scientific attention, and promise that with further deeper research chitosan may have a much better development and successful application.

In addition to macromolecular substances, some with low molecule weight such as saponins and carotenoids also have great influence, which will be discussed in the following content.

### 3.4. Saponins

Saponins are interesting bioactive compounds with marine animal origin. The sea cucumber saponins, for example, are triterpenoid glycosides composed of triterpenoids and carbohydrate parts [105]. The effects of saponins on the hyperlipidemia have been extensively studied [106]. Results show that saponins from the sea cucumber dose dependently reduce the serum levels of TC and LDL-C, whereas increase the levels of HDL-C in HFD rats. It was further shown that they lowered the ApoE-/-mice serum levels of TG and TC, increased the level of HDL-C, enhanced the liver expression of ABCG5/8 as well as promoted liver cholesterol efflux, which contributed to the decrease of free cholesterol in the liver. The effect of the sea cucumber saponin was even stronger than that of ginsenoside, as it significantly reduced the liver and serum lipid levels of HFD mice, inhibited lipid synthesis, accelerated liver fat oxidation and successfully reduced body weight [107]. Additionally, it was shown that the liposomes of certain types of sea cucumber saponins on HFD mice, compared with the common form of sea cucumber saponins, exhibited improved activities in weight reduction and anti-hyperlipidemia action. Simultaneously, these saponins reduced the inflammation of adipose tissues and improved insulin resistance [108]. The exploration of the hypolipidemic effects of saponins and their application forms as well as the further understanding of their mechanisms are essential for the development and utilization of related drugs.

### 3.5. Astaxanthin

**Figure 5:**
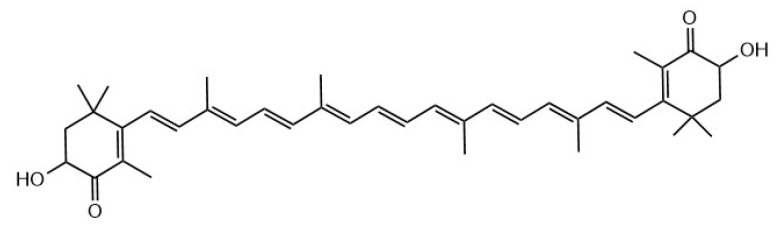
The structure of astaxanthin [114].

Astaxanthin is a red lutein or oxygenated carotenoid known for its powerful antioxidant activity whose structure is shown in Figure 5 [109]. Studies have found that astaxanthin can decrease the TC, TG and LDL-C of hyperlipidemic mice [110]. Others have shown that astaxanthin significantly reduced blood serum levels of TG, TC and LDL, and the level of TG in the liver, as well as increased HDL levels. Furthermore, it reduced the levels of serum ALT and AST in HFD mice [111]. Researchers explored the effects of astaxanthin on the lipid metabolism in live of mice fed HFD, and have found that astaxanthin effectively reduced TC and LDL-C, increased HDL-C, and improved lipid metabolism when compared with the HFD group [112]. Others have got that astaxanthin reduces hepatic lipid accumulations in HFD fed C57BL/6J mice via activation of PPAR alpha and inhibition of PPAR gamma and Akt [113]. Different from polysaccharides and peptides, the structure of astaxanthin has been fully understood, and its separation and purification are relatively easy, which provides convenience for the research of astaxanthin. At present, there are few studies on the lipid lowering function of astaxanthin, and its specific mechanism remains to be further explored.

We have organized the animal-derived marine products with hypolipidemic effects in recent years, as shown in Table 2.

## 4. Secondary Metabolites and Other Marine-Derived Products

In addition to the above, some other marine products exhibit strong lipid-lowering effects. The furanone isolated from *Fungus setosphaeria sp* SCSIO41009 was shown to significantly reduce lipid accumulation in ox-LDL induced RAW 264.7 cells [118]. It was shown that the cellular models used in this study demonstrated a significant reduction in its TG content, and an increase in the expression of PPAR-alpha and ABC transporters. In HepG2 cells, furanone efficaciously reduced the lipid accumulation caused by oleic acid, improved LDLR, ABCG5, ABCG8 and PPAR-alpha expression and reduced the expression of SREBP-2. Subsequent results showed that LXR-alpha and PPAR-alpha could be targeted to exert their hypolipidemic effect [118]. Isolated from *Fungus xylaria sp.* (No.2508), xyloketal B, lowered the liver tissue lesions, reduced lipid accumulation in the blood and liver and improved the expression of CPT1A as well as downregulated the expression of SREBP-1c and its downstream ACC1, ACL and FAS in mice fed HFD. Cell experiments also proved that xyloketal B reduced lipid accumulation in HepG2 cells. These results indicate that xyloketal B regulates lipid metabolism through the SREBP-1c pathway [119]. Since the discovery of antibiotics, the secondary metabolites of fungi have been an important source of drug development. At present, there are few studies on the secondary metabolites of marine fungi with hypolipidemic effects. With the development and deepening of relevant research in the field, new lipid-lowering drugs will emerge gradually.

Apart from that, there are other marine products exhibiting hypolipidemic ability. Many studies have been conducted on the beneficial effects of deep sea water (DSW). DSW increases mitochondrial synthesis in 3T3-L1 pre-adipocytes, increases gene expression of PGC1-alpha, NRFI and TFAM, upregulates oxidative activity of cytochrome C and phosphorylation level of AMPK, thereby exerting lipid lowing effects [120]. Researchers got that DSW decreased intracellular triglyceride and glycerol-3-phosphate dehydrogenase activity in 3T3-L1 adipocytes and inhibited the differentiation of adipocyte, adipogenesis and expression of adipose cytokines [121]. It is proven that DSW increases the levels of fat hydrolysis and oxidation in a dose-dependent manner. In animal experiments, it significantly reduces body weight, liver, adipose tissue, hepatic triglycerides and cholesterol, and serum parameters. DSW can also improve fecal output of total lipids, triglycerides, and cholesterol, decreases serum TG and TC levels, downregulates the expression of AMPK, PPAR-alpha, CPT-1 and the ACO, thus exerting hypolipidemic effects. The ability of DSW in reducing serum levels of ALT is shown in HFD fed mice [121]. Others showed that the DSW reduces the level of TC, downregulates the expression of HMGCR, upregulates AMPK phosphorylation, and decreases the synthesis of cholesterol in HepG2 cells, Meanwhile, it could also upregulate the expression of LDLR, PPAR-alpha, SREBP-1alpha and SREBP-2, and can lower the expression of the proprotein convertase subtilisin/kexin type 9 (PCSK9) [122]. The fact that DSW reduced the levels of TC and LDL-C whereas increased the level of HDL-C, decreased the expression of fatty acid synthase and SREBP-1c in the blood of rats fed with a HFD and enhanced the expression of LDLR, thus exerting a lipid lowering effect was also showed [123]. To our surprise, DSW has a significant lipid-lowering effect. Although the mechanism remains to be further determined, current research undoubtedly provides a new idea for the development of lipid-lowering drugs on the basis on the DSW. A possible explanation of its beneficial activities is the fact that the DSW has a low temperature and high purity. It is also rich in nutrients due to sparse photosynthesis and little number of marine organisms at this depth. Some of the beneficial elements of DSW include magnesium, calcium, potassium, chromium, selenium, zinc, and vanadium. Research has proven that DSW can help overcome health problems, especially related to lifestyle-associated non-communicable diseases such as cardiovascular disease, diabetes, obesity, cancer, and skin problems.

We have organized the other marine products with hypolipidemic effects in recent years, as is shown in Table 3.

## 5. Conclusions and Future Outlooks

Nowadays, cardiovascular diseases have seriously threatened people’s lives and have become the leading destroyer of health. Atherosclerosis is a common cause of cardiovascular diseases. As a metabolic disorder characterized by hypercholesterolemia, hypertriglyceridemia and decreased HDL, hyperlipidemia is closely related to atherosclerosis. Due to the complex pathological mechanism of hyperlipidemia involving multiple systems, it is difficult for common drugs to achieve satisfactory results. Because of the high efficiency and low toxicity, natural products have great potential in the prevention and treatment of hyperlipidemia.

With the development and utilization of marine living resources in recent decades, the consumption and demand of marine products have gradually increased. Compared with terrestrial organisms, marine ones live in more complex environments and therefore contain many bioactive substances. Recently, extensive research has confirmed the lipid-lowering effect of many marine nature products with different origin.

In this paper, the application of algal and animal marine products in lipid reduction is reviewed. At present, algae have been studied most extensively and therefore produced many valuable research results. These results show that many algal polysaccharides have lipid-lowering activity. Due to the diversity and heterogeneity of the structure of seaweed polysaccharides, it is challenging to study the structure, which also hinders the development and utilization of algae polysaccharides. Compared with other marine products, algae are much more suitable for large-scale production, thus effectively reducing the acquisition cost of bioactive polysaccharides. In view of the fact that algal polysaccharides change with their living environment, homogeneity has become a noteworthy problem. Until now, some polysaccharides from seaweed have emerged in the field of cosmetics, which gradually expands the application prospect of seaweed polysaccharides.

Other products of marine sources, such as bioactive peptides have good bioactivity despite relatively high cost, and have shown great results in experiments studying their biological activities, attracting the attention of many researchers. Besides, the hypolipidemic effect of secondary metabolites of fungi and deep sea water is very innovative and recently has revealed worthy results, which provide a new idea for the development of lipid-lowering drugs on their basis.

Though most marine products are relatively safe, the potential for adverse toxicological side effects appears to be present, which might be relevant if higher doses are utilized. Given the fact that some lipid lowering-drugs have detrimental effects, such as torcetrapib, the possible harmful effects should also be paid attention to during drug development.

Apart from lipid-lowering effects, marine products also have biological activities such as lowering blood pressure, anti-tumor and immunity-regulating effects. The development and utilization of marine products is of great significance for their high-precision processing and high-value utilization. With a deeper understanding of relevant studies, marine bioactive substances will be gradually applied in food and pharmaceutical industries, which will further expand the areas for their application and make a great contribution to human health.

## Figures and Tables

**Table 1:** Algae products with lipid lowering effect.

Name	Source	Experimental Model	Index	Mechanism	Ref
Porphyran	*Pyropia yezoensis*	Hyperlipidemia mice	TC, TG, LDL-C, HDL-C	To be determined	[29]
Sulfated polysaccharides	*Padina tetrastromatica*	Isoproterenol Induced myocardial infarction rat model	TC, TG, LDL-C, HDL-C	Regulate the expressions of SREBP-2 and LDL-R	[30]
Fucoidan A3	*Ascophyllum nodosum*	Hyperlipidemia mice	TC, TG, Fat pad index	Enhance RCT-related genes expression	[38]
Fucoidan	*Ascophyllum nodosum*	Hyperlipidemia mice	TC, TG, Fat pad index	Enhance RCT-related genes and proteins expression	[39]
Fucoidan	*Fucus vesiculosus*	Hyperlipidemia mice	TC, TG, LDL-C, HDL-C	Regulate the expression of key enzymes of cholesterol and triglyceride syntheses	[41]
Fucoidan A2	*Ascophyllum nodosum*	Hyperlipidemia mice	TC, TG, HDL-C, Fat pad index	Modulate RCT-related protein expression	[64]
Fucoidan	*Kjellmaniella crassifolia*	Hyperlipemia rats	TG	Inhibit cholesterol and aliphatic acid synthesis, accelerate the oxidation of aliphatic acid	[65]
Fucoidan	*Sargassum wightii*	Hyperlipidemia mice	TC	Inhibit HMGCoA reductase activity, improve LCAT, HL, LPL activity	[66]
Fucoidan	Brown algae	Apolipoprotein E-deficient mice	TC, TG, LDL-C, HDL-C	Induce LPL activity, inhibit the effects of inflammation and oxidative stress	[67]
Sodium alginate	*Turbinaria ornata (Turner) J.Agardh*	Diabetic rats	TC, LDL-C, HDL-C	Increase fecal excretion of cholesterol	[42]
Sodium alginate and amidated sodium alginate	Brown algae	Hyperlipemia rats	TC, TG	Increase fecal excretion of cholesterol and coprostanol	[43]
Calcium alginate	Brown algae	Hyperlipemia rats	TC	Enhance fecal excretion of bile acid	[44]
Ulvan	*Ulva pertusa*	Hyperlipidemia mice	TC, TG, LDL-C, HDL-C	Protect against the liver damage of oxidative stress	[47]
Ulvan	*Ulva pertusa*	Hyperlipidemia mice	TC, TG, LDL-C, HDL-C	Improve hypolipidemic activities	[48]
Carrageenan	*Kappaphycus alvarezii*	Hyperlipidemia mice	TC	Regulate genes in lipid metabolism	[49]
Carrageenan	*Eucheuma spinosum*	Hyperlipidemia mice	TC, TG, LDL-C, HDL-C	Increase intestinal content, inhibit intestinal mucosal absorption, accelerate peristalsis in the small intestine	[50]
Fucoxanthin	*Laminaria japonica*	Hyperlipidemia mice	TC, TG	Regulate the expression of IRS-1/PI3K/AKT and AMPK signaling pathway	[55]
Fucoxanthin	*Phaeodactylum tricornutum*	Hyperlipidemia mice	TG	Increase enzymatic activity of lipoprotein metabolism key enzymes	[59]
Fucoxanthin	Algae	Obese people	TG	Inhibit lipid accumulation	[60]
Phlorotannins	*Ecklonia cava*	Hypercholesterolemia people	TC, LDL-C	Inhibit lipid accumulation	[62]
Diphlorethohydroxycarmalol	*Ishige okamurae*	Hyperlipidemia mice	TG, LDL-C, HDL-C	Inhibit lipid biosynthesis	[63]

**Table 2:** Marine products of animal-derived with hypolipidemic effect.

Name	Source	Experimental Model	Index	Mechanism	Ref
Protein hydrolysates	Goby fish	Hyperlipidemia rats	TC, TG, LDL-C	Inhibit lipid accumulation	[74]
Fish protein	Sardine	Hyperlipidemia rats	TC, TG	Reverse cholesterol transport	[75]
Protein hydrolysates	Smooth hound	Hyperlipidemia rats	TC, TG	Not specified	[77]
Protein hydrolysates	*Octopus vulgaris*	Hyperglycemic rats	TC, TG, LDL-C	Inhibit lipid accumulation	[78]
Protein hydrolysates	*Sardinella aurita*	Hyperlipidemia rats	TC, TG, LDL-C, HDL-C	Decrease the pancreatic lipase activity	[76]
Collagen peptide	Tuna	3T3-L1 preadipocytes	TC, TG	Inhibit adipocyte differentiation	[73]
Vanadium-binding proteins	*Squirt Halocynthia*	3T3-L1 Adipocytes	TC, TG	Decrease adipogenesis	[115]
DHA	Fish oil	Hyperlipidemia mice	TC, TG	Inhibit lipogenesis	[87]
EPA/DHA	Deep-sea fish oil	Hyperlipidemia rats	TC, TG, LDL-C	Regulate the response level to oxidative stress, improve expression level of the SIRT1 and PPAR-a proteins	[88]
DHA/EPA	Starfish Oil	Hyperlipidemia mice	TC, TG, LDL-C	Improve lipid metabolism	[89]
EPA	*Cucumaria frondosa*	Hyperlipidemia mice	TG, HDL-C	Suppress lipid accumulation	[116]
Phospholipid	*Cucumaria frondosa*	Hyperlipidemia mice	TC, TG, LDL-C	Suppress hepatic fatty acid synthesis, enhance hepatic fatty acid B-oxidation	[90]
Sulfated polysaccharides	Abalone gonad	Hyperlipidemia mice	TC, TG, LDL-C, HDL-C	Inhibited fat accumulation	[91]
Sulfated polysaccharides	*Stichopus japonicus*	Hyperlipidemia mice	TC, TG, LDL-C	Modulate the gut microbiota, improve microbial metabolites and gut tissue	[92]
Fucoidan	*Pearsonothuria graeffei, Isostichopus badionotus*	Hyperlipidemia rats	TC, TG, LDL-C, HDL-C	Improve lipid metabolism	[93]
Fucoidan, fucosylated chondroitin sulfate	*Pearsonothuria graeffei, Isostichopus badionotus*	Hyperlipidemia rats	TC, TG, LDL-C, HDL-C	Inhibit pancreatic lipase	[96]
Chitooligosaccharide	Commercial procurement	Hyperlipidemia mice	TC, TG, LDL-C	Decrease the uptake of FFAs and triglyceride synthesis	[102]
Chitooligosaccharides	Commercial procurement	Hyperlipidemia rats	TC, TG, LDL-C, HDL-C	Improve lipid metabolism	[103]
Chitooligosaccharides	Snow crab	HepG2 cells, Hyperglycemic rats	TC, TG, LDL-C	Regulate HMGCR, improve lipid metabolism	[104]
Saponins	Sea cucumber	Hyperlipidemia rats	TC, TG, LDL-C, HDL-C	Enhance RCT-related genes and proteins expression	[106]
Saponins	*Pearsonothuria graeffe*	Hyperlipidemia mice	TC, TG, LDL-C, HDL-C	Inhibit lipid synthesis, accelerate lipid beta-oxidation	[107]
Saponins	Sea cucumber	Hyperlipidemia mice	TC, TG, LDL-C, HDL-C	Improve lipid metabolism	[108]
Echinoside A	*Pearsonothria graeffei*	Chow fed mice	TC, TG	Improve lipid metabolism	[117]
Astaxanthin	Commercial procurement	Nonalcoholic fatty liver disease	TC, TG, LDL-C, HDL-C	To be determined	[111]
Astaxanthin	Commercial procurement	Hyperlipidemia mice	TC, TG, LDL-C, HDL-C	Improve lipid metabolism	[112]
Astaxanthin	Commercial procurement	Hyperlipidemia mice	TC, TG, LDL-C, HDL-C	To be determined	[110]
Astaxanthin	Commercial procurement	Hyperlipidemia mice	TC, TG	Activate PPAR alpha and inhibit PPAR gamma and Akt	[113]

**Table 3:** Some other products with lipid-lowing effect of marine-derived.

Name	Source	Experimental Model	Index	Mechanism	Ref
Furanone	*Fungus setosphaeria* sp	RAW 264.7 cells	TC, TG	Upregulate PPARα	[118]
Xyloketal B	*Fungusxylaria* sp	Hyperlipidemia mice	TC, TG, LDL-C	Reduce lipid accumulation	[119]
Cube natural sea salt	Sea	3T3-L1 adipocytes, Hyperlipidemia mice	TC, TG, LDL-C	Reduce lipid accumulation, regulate the beta-oxidation, lipolysis	[124]
Deep sea water	Sea	3T3-L1 preadipocytes	TC, TG, LDL-C	Improve lipid metabolism	[120]
Deep sea water	Sea	3T3-L1 preadipocytes, Hyperlipidemia rats	TC, TG, LDL-C	Improve lipolysis and fatty acid oxidation	[121]
Deep sea water	Sea	HepG2 cells	TC, TG, LDL-C	Induce LDLR and ApoA1 transcriptions, inhibit PCSK9 mRNA expression	[122]
Deep sea water	Sea	Hyperlipidemia rats	TC, LDL-C, HDL-C	Enhance LDLR expression, suppress fatty acid synthase and SREBP-1c expression	[123]

## Abbreviations

**ABC**	ATP-binding cassette transporter
**ACC**	acetyl-CoA carboxylase
**ALT**	alanine aminotransferase
**AMPK**	Adenosine 5′-monophosphate-activated protein kinase
**aP2**	adipocyte protein 2
**AST**	aspartate aminotransferase
**CAT**	catalase
**C/EBP**	CCAAT/enhancer binding protein
**CVD**	cardiovascular diseases
**CYP7A1**	cholesterol 7alpha-hydroxylase A1
**DHA**	docosahexaenoic acid
**DSW**	deep sea water
**EPA**	eicosapentaenoic acid
**GSH-Px**	glutathione peroxidase
**HDL-C**	high-density lipoprotein cholesterol
**HMGCR**	3-hydroxyl 3-methyl glutaryl coenzyme A reductase
**LCAT**	Lecithin: cholesterol acyltransferase
**LDL-C**	low-density lipoprotein cholesterol
**LDLR**	LDL receptor
**LXR**	Liver X receptor
**NCD**	noncommunicable diseases
**NPC1L1**	Niemann-Pick C1 like 1
**PCSK9**	proprotein convertase subtilisin/kexin type 9
**PGC1**	PPARγ coactivator 1α
**PPAR**	peroxisome proliferator-activated receptor
**PUFA**	polyunsaturated fatty acids
**SMYD3**	SET and MYND domain containing 3
**SOD**	superoxide dismutase
**SR**	scavenger receptor
**SREBP**	sterol-regulatory element binding protein
**TC**	total cholesterol
**TG**	triglyceride
**UCP**	uncoupling protein
